# The Importance of Brain Banks for Molecular Neuropathological Research: The New South Wales Tissue Resource Centre Experience

**DOI:** 10.3390/ijms10010366

**Published:** 2009-01-23

**Authors:** Irina Dedova, Antony Harding, Donna Sheedy, Therese Garrick, Nina Sundqvist, Clare Hunt, Juliette Gillies, Clive G. Harper

**Affiliations:** 1 Schizophrenia Research Institute, Sydney, NSW 2010, Australia. E-Mails: nsundqvist@med.usyd.edu.au (N. S.); jgillies@med.usyd.edu.au (J. G.); 2 The New South Wales Tissue Resource Centre, Discipline of Pathology, The University of Sydney, NSW 2006, Australia. E-Mails: aharding@usyd.edu.au (A. H.); donnas@med.usyd.edu.au (D. S.); thereseg@med.usyd.edu.au (T. G.); chunt@med.usyd.edu.au (C. H.); cliveh@med.usyd.edu.au (C. G. H.)

**Keywords:** Human, brain bank, schizophrenia, alcohol, postmortem, molecular neuropathology, genome, proteome, receptor binding, clinical characterization

## Abstract

New developments in molecular neuropathology have evoked increased demands for postmortem human brain tissue. The New South Wales Tissue Resource Centre (TRC) at The University of Sydney has grown from a small tissue collection into one of the leading international brain banking facilities, which operates with best practice and quality control protocols. The focus of this tissue collection is on schizophrenia and allied disorders, alcohol use disorders and controls. This review highlights changes in TRC operational procedures dictated by modern neuroscience, and provides examples of applications of modern molecular techniques to study the neuropathogenesis of many different brain disorders.

## 1. Introduction

Molecular age technology represents unique opportunities to look at what we know about normal and abnormal brain structure and function from a new angle. Advances in the fields of genomics and proteomics offer new ways to study the pathophysiology of illnesses such as schizophrenia, alcohol use disorders, brain tumors, inflammatory and degenerative brain disorders. Despite significant advances in other methodological approaches, access to postmortem human brain tissue is vital for further progression of our knowledge on molecular neuropathology. This review highlights how one brain bank has developed to help scientists learn more about molecular neuropathology of diseases. With joint efforts from researchers, clinicians, patients, granting bodies and the general public, the brain bank is a powerful resource enabling new discoveries at the frontiers of neuroscience.

The last two decades have been marked by drastic developments in high throughput approaches in molecular neuroscience. The ‘neurogenomics’ and ‘neuroproteomics’ fields have evolved such that we now have the ability to screen and compare whole genomes and proteomes of specific brain regions [1]. Careful selection of study cohorts and attention to the quality markers of tissue samples can have a tremendous impact on the outcomes from any research based on molecular techniques. To be able to apply these new technologies to study the human brain, scientists require access to multiple samples of well characterized postmortem human brain tissue preserved by freezing at ultra-low temperatures (–70 ºC to –85 ºC). However, until recently, the majority of hospital and university collections acquired mostly formalin-fixed brain tissue used primarily for diagnostic purposes. The urgent need for non-fixed frozen postmortem brain material for research is apparent.

A high worldwide prevalence of neurodegenerative diseases, alcohol use disorders and other psychiatric conditions including schizophrenia [2], has placed these illnesses at the front edge of neuroscience research. This trend has resulted in an increased demand for human brain tissue samples [3–5]. The acquisition of donors with an alcoholic or a psychiatric background, in particular, presents specific challenges. These patients are very often single, unemployed, live alone with minimal to no contact with their next-of-kin (NOK), and are at high risk of going missing or becoming homeless. Therefore, following the death of such patients, it is often very difficult to locate the NOK, thereby limiting the possibility of obtaining appropriate consent within an acceptable time frame. In addition, there are specific difficulties associated with obtaining satisfactory clinical information for neuropsychiatric characterization and diagnosis [5]. Retrospective characterization of cases requires additional resources and can be a limiting factor affecting the validity of any investigation using these cases.

The availability of ‘normal control’ cases for comparative studies is often a limiting factor [3]. Tissue collections that originate from hospital autopsies are primarily focused on illness and do not often contain unaffected normal control cases, resulting in a shortfall. With molecular techniques requiring large numbers of cases to reach statistical significance, and a continuing decline in hospital autopsies around the world [6] the situation is made even more desperate for tissue banks. This has generated a growing interest in the improvement of human brain tissue banking practices, and the establishment of pre-mortem brain donor programs [3, 6].

## 2. The New South Wales Tissue Resource Centre (TRC)

In 1994 the Australian government provided funds to establish a number of brain banks in different states to encourage more studies in the important field of mental health disorders, one of which was the New South Wales Tissue Resource Centre (TRC). It has since grown from a small collection of mostly formalin-fixed human postmortem brain tissue samples used for intramural research projects, into one of the leading brain banking facilities globally. The TRC provides both fixed and freshly frozen human brain tissue for Australian and international investigators. The collection focuses on schizophrenia and allied disorders, and diseases related to alcohol use. Importantly, the TRC also acquires ‘normal control’ cases, an invaluable comparison group. For all cases, inclusion and exclusion criteria are stringently applied [7]. Since its establishment, the TRC has supported around 280 tissue requests from 120 different neuroscience research groups worldwide. The TRC maintains strong ethical principles, and policies and procedures have been developed to ensure that they reflect community standards and meet legal requirements. This includes the complex issue of obtaining consent from donors and their families. An independent Scientific Board reviews all tissue requests from researchers focusing on the scientific merit and feasibility of the projects.

At present, the TRC has more than 550 cases available for research use. These include over 120 well characterized controls, 116 cases with alcohol use disorder and related pathology, 90 psychiatric cases - including schizophrenia and schizoaffective disorders, and 44 motor neuron disease cases. There are also cases with Alzheimer type pathology, ischemic and vascular pathology, brain tumors and cases with mixed and rare neuropathology.

The main source for the collection of cases is ‘at death’ donations by the NOK through the Department of Forensic Medicine in Sydney, Australia. Recent figures reviewed over a five year period (2002–07) reveal that 54% of the NOK agreed to donate the brain tissue of their relative (unpublished observations). The most common reasons offered for the donation were altruism and compliance with the known wishes of the deceased. In 1998, due to decreased autopsy rates and to obtain prospectively collected detailed information about donors, the first of our pre-mortem brain donor programs was established. The ‘Using Our Brains’ program invites people with no neurological or psychiatric illness (controls) to consent to brain donation after death. In 2002 the ‘Gift of Hope’ brain donor program was launched to recruit people with psychiatric illnesses, especially schizophrenia.

## 3. Changing nature of tissue requests and operational protocols at the TRC

At autopsy, the brain is processed according to a standardized protocol [7], which allows the neuropathological examination of each case, as well as ensuring that both fresh-frozen and formalin-fixed tissue is available for research. Collection procedures and dissection protocols employed by the TRC have developed over time, based on feedback from neuroscientists using the tissues for research. Recent improvements in research methodologies have dictated changes in the requirements for a postmortem human brain tissue sample. In the 1990’s, 85% of projects supported by the TRC used formalin-fixed tissue, with under 10 cases per project requested on average. In the last five years (2003–08), however, over 80% of tissue requests received by the brain bank were for non-fixed fresh frozen brain tissue, with 20 to 30 cases per disease group per project requested on average. Simultaneously, the size of tissue samples required has decreased dramatically from 5–10 grams per block in the 1990’s down to 0.1 gram of tissue required for today’s studies. To illustrate this point, in 2007–08 the majority of the supported projects employed genomics (51%) and proteomics (24%) methodologies, all of which required: (a) large cohort sizes; (b) fresh frozen tissues; (c) highly specific areas of the brain (e.g. grey matter from the Broadman area 9); and (d) an average tissue sample size of 0.1–0.2 grams.

Therefore, over the years, the dissection protocol at time of donation, has changed from a simple procedure where only a few tissue blocks (if any) were frozen, to a state-of-the-art highly specialized and standardized dissection protocol with over 70 specific brain regions dissected freshly, and frozen separately [7]. Such protocols maximize the availability of specific frozen brain regions for research and minimize the handling and potential thawing of frozen tissue when it is being prepared for the researcher. Detailed documentation with photographic images of tissue blocks taken at the time of dissection, are invaluable in assessing tissue availability and assisting staff when the request is being processed. The importance of appropriate qualifications and provision of ongoing training for staff members of the TRC cannot be overstated.

The increased specificity of frozen tissue requests introduces an additional challenge for brain banking. Using highly sensitive molecular biology techniques, researchers now aim for very specific and macroscopically small brain regions. In contrast to gross anatomical regions, e.g. ‘hippocampus’, in order to address relevant biological questions, neuroscientists now often require specific brain regions, e.g. ‘hippocampal CA1 region from the anterior hippocampus at the level of the uncus’. In this respect, the contribution of the brain bank to a given research project can go significantly beyond just providing the tissues. Highly qualified staff thoroughly evaluates every tissue request to ensure that the appropriate brain area is available for the study cohorts, and most importantly that all samples provided are comparable between and within the study cohorts. Processing of such tissue requests can take considerable time and effort, but it is a key component for the success of any well-planned investigation. Providing tissue for the multiple tissue requests received, inevitably leads to ever-changing availability of the different brain regions stored at the brain bank at any given time. Detailed tissue database records including digital photographs are essential so that tissue availability can be readily retrieved from a database reducing unnecessary handling of frozen material.

## 4. Tissue integrity and clinical information quality assurance

New molecular techniques dictate a need for the highest standards in the quality of the samples. There are numerous confounding pre- and post-mortem factors that are thought to play a role [8–12]. The most important factor affecting gene expression in postmortem human brain tissue is believed to be the total RNA quality [9]. In addition, other factors which must be considered are tissue pH, postmortem interval, age, agonal state and smoking history [8, 9]. These factors must be taken into account when selecting cases for the study cohorts. From the brain bank’s point of view, this emphasizes two main issues: tissue quality assurance (the tissue integrity domain) and case selection and characterization (the clinical domain).

### 4.1. Tissue integrity domain

The TRC measures brain tissue pH as part of the standard brain banking protocol [7, 13]. This data together with the results of detailed macroscopic and microscopic neuropathological examination is made available to researchers. The total RNA yield and quality data, however, is obtained from researchers who are using the tissue. RNA integrity may vary between the different brain regions even within the same case [8, 9]. It may also vary within the same region over time following tissue handling and due to temperature fluctuations when the same tissue block is used to obtain samples for multiple requests. Thus, the feedback from researchers using these tissues is very useful in establishing the best quality tissue. It also confirms the efficacy of the existing TRC protocols. Such a cost-effective approach generates a comprehensive dataset for every case, on the most valuable and relevant brain regions that otherwise would be not assessed. Importantly, RNA integrity is assessed by a number of separate and independent research laboratories with expertise in the field, and the integrity assessment is performed longitudinally. This not only gives us objective feedback on tissue integrity, but also limits any potential artifacts or cases of mistaken poor RNA extractions. Taken together, the brain tissue pH, neuropathological diagnosis and RNA integrity data allow the TRC to select high quality research material.

Based on evaluations received from researchers in the 2007–08 period, 96% of researchers commented on the high quality of the frozen tissue and RNA integrity from the samples provided by the TRC. Notably some of these samples had been stored for up to 12 years without degradation of tissue quality. However, the preparation of synaptosomes with intact morphology from frozen tissues is compromised (unpublished observations) due to the damaging effects of rapid freezing on membrane structures [14]. The cases with lower tissue integrity that are excluded from mainstream research are also precious and in high demand by researchers for the development and testing of new methodologies. The TRC assists with 10–20 requests for ‘trial’ tissue annually.

### 4.2. Clinical domain

The TRC operates with standardized clinical information collection protocols that have evolved over many years. Humans are ‘difficult’ subjects to study: we cannot control their environment, diet, lifestyle, habits, the illnesses they may develop, how they are treated, or ultimately how they die. Researchers need to be aware of the multitude of pre-mortem factors that can influence their data. The clinical domain therefore provides another significant area of input by the TRC into supported research projects. In-depth retrospective clinical and lifestyle characterization of cases following the death of a donor is a time-consuming exercise and requires specialist staff. However, without adequate clinical information, it is not possible to select and match cases appropriately for various study cohorts.

A sound, reliable psychiatric diagnosis is critical for brain banking facilities such as the TRC, which aims specifically to support research into psychiatric illnesses and alcohol use disorders. All psychiatric or alcohol use disorder diagnoses are confirmed retrospectively using the Diagnostic Instrument for Brain Studies – Revised (DIBS-R) [15]. The DIBS is semi-structured instrument designed specifically for post-mortem psychiatric assessment using medical records and informants where available. This instrument is compliant with the Diagnostic and Statistical Manual of Mental Disorders (DSM-IV) [16] and has demonstrated reliability [17].

The DSM-IV diagnosis assigned to each case (derived from the DIBS) is based on a detailed and standardized clinical assessment summary created by specially trained staff with a background in psychiatry and/or psychology. The structure of this summary readily allows assessment of diagnostic inter-rater reliability. Control cases are characterized using the same process as employed for psychiatric cases, to ensure appropriate use of each case in a comparison group. A recent comprehensive assessment of the reliability of postmortem TRC psychiatric diagnoses, relative to lifetime (ante-mortem) diagnoses from medical records, showed moderate to excellent inter-rater reliability for most cohorts. There was sufficient disagreement, however, particularly in relation to the schizoaffective disorder cohort, to highlight the importance of a standardized approach to psychiatric diagnosis. Also illustrated in this study was the importance of accurate medical record keeping at a symptom-based level [18].

The initial body of information collected on cases in the mid 90’s rarely included comprehensive lifestyle information. Now, however, the TRC specifically aims to collect information on factors such as medications, illicit drug use, and alcohol and tobacco use for every case. During the last three years, all requests for tissues from psychiatric cases sought detailed information - not only regarding the age of disease onset and duration of illness, but also on the antipsychotic drug treatments prescribed, including the predominant type of antipsychotics used, and an estimate of the lifetime chlorpromazine equivalent medication dosages. Details of tobacco and alcohol use in both the study and control groups are also sought by almost 100% of researchers requesting tissue. Along with the cause of death, other autopsy findings such as toxicology reports and liver pathology are also frequently asked by researchers. The ability to operate with these facts and to cross-correlate research findings with clinical and lifestyle information is a powerful tool in molecular type studies [19, 20].

## 5. Evolution of the TRC

Brain banking is necessarily a long-term project and requires continued commitment from institutions and staff, as well as considerable infrastructural financial support. The numbers of cases collected each year have grown from just over 25 cases per year in the 90’s to ~65 in 2008. In the first five years (1994–99) the TRC fulfilled a total of 34 tissue requests, almost all (~90%) of which were from researchers associated directly with the brain bank. The majority of these early projects were related to alcohol and schizophrenia research. By comparison, during the last five years (2003–08), the TRC has fulfilled on average 34 research projects each year. In 2008, approximately one third of requests arose from researchers directly associated with the brain bank, while the remainder came from external research groups - including 25% of requests from international researchers. While the ‘traditional’ research focus on schizophrenia (39% of requests in 2008) and alcohol-related disorders (31%) is maintained, there are a growing number of research projects in other fields (30%). The latter group includes projects on neurodegenerative diseases, as well as basic neuroscience investigations into brain structure and function. The most requested case types from the TRC for these studies are controls (see below). With the publication of research outcomes from studies using TRC-sourced tissues, there is mounting demand for postmortem human brain tissue from our facility. Although the TRC strongly preserves its focus on schizophrenia and allied disorders, as well as alcohol-related diseases, the expansion of its role beyond this focus is evident. Fortunately, there is strong public interest in brain donation, and the number of people expressing their willingness to consent pre-mortem is growing.

The maintenance of brain banking resources and pre-mortem brain donor programs comes at a high cost. Each brain successfully banked and made available for research costs at least A$15,000 or more. This includes costs associated with employment and training of administrative, technical, clinical and research staff, as well as neuropathologists. Costs for body transport and technical assistance for autopsies are often forgotten. There are other costs associated with maintenance of storage facilities and laboratories, consumables, databases and web-based information systems. Funding is difficult to obtain from competitive granting bodies since it is not ‘project driven’ research. Thus, brain banks are largely reliant upon government bodies to provide the infrastructural support. Brain banks should be seen in the same light as preventative medicine programs. Given the emerging technologies that can be applied to appropriately-stored human brain tissue and the burden of mental health disorders in our society we must ensure that researchers can be provided with high quality human brain tissue. The planning and support that brain banking receives today will determine how well future progress in molecular neuroscience can be provided for.

## 6. Australian Brain Bank Network

Brain banks have been established in Australia and throughout the world to ensure a diverse collection of brain tissues. The recruitment and collection regimes for each of these banks are often developed independently, usually in order to facilitate the collection of cases for use by a bank’s own researchers. In Australia, brain donor programs and brain banks have been established in five state capital cities. In 2003, the National Health and Medical Research Council of Australia funded a grant enabling these brain banks to liaise with one another as the Australian Brain Bank Network (ABBN). The ABBN ensures the availability of a greater number and range of human brain tissues for researchers (http://www.nnf.com.au/platforms/abbn/). By 2008, a total collection of more than 1,800 cases has been made available for research. Each ABBN brain bank employs standardized protocols, so that tissue provided by any site is comparable in its preparation and storage methodologies. Compared with published methodologies of other international brain banks, the ABBN operations are in line with the best known practices [3, 21–23].

## 7. Specific examples of molecular neuropathological studies using TRC tissues

**Figure 1.**
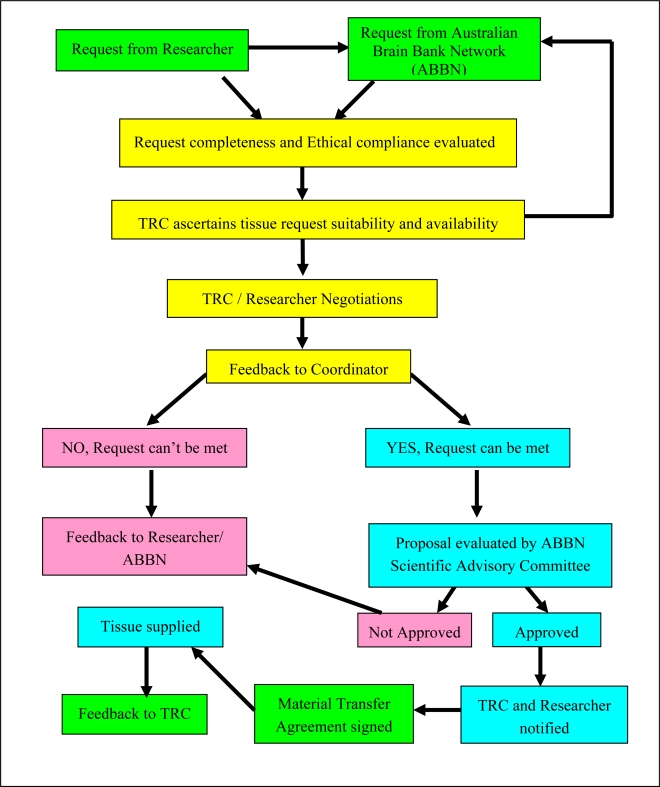
A flow-chart of the tissue request process at the TRC. Colors represent different domains in the request process: green – a researcher’s domain; yellow – the TRC domain; blue – a successful tissue request; and red – an unsuccessful tissue request.

Any tissue request received by the TRC undergoes a thorough review process (Figure 1) by a Scientific Advisory Committee, consisting of independent scientists and clinicians with proven expertise in related research areas, as well as the TRC directors and representatives. The review process that usually takes two to three weeks includes: (a) scientific merit; (b) proof of methodology; (c) track record; (d) ethical compliance; (e) tissue availability; (f) potential conflict of interest; and (g) justification of the region, type and amount of human brain tissue requested.

### 7.1. Schizophrenia

The pathophysiology of schizophrenia involves the alterations in neurotransmitter systems such as dopamine, glutamate, GABA-ergic, serotonergic and cholinergic to name the few. Although the diagnosis of schizophrenia is primarily phenomenological, the neuroanatomical changes are believed to be widespread, largely involving impaired neural circuitry between and within prefrontal and temporal cortices, limbic system and the midbrain. The cohorts collected by the TRC have been successfully used in studies aiming to understand specific molecular mechanisms or systems responsible for schizophrenia.

Proteomics allows changes in the global protein expression and modification to be investigated and allows us to picture the translation of genomic information together with co- and post-translational modifications [24]. The brain proteome profiles of two regions, the anterior cingulate cortex (ACC) and the genu of the corpus callosum (CÑ), from the schizophrenia and control cohorts have been compared [24–26]. For such studies, which screen large pools of proteins, it is imperative to eliminate any false positives due to pre- or post-mortem factors or due to the brain proteome regional differences. By using exactly the same cohorts in three different proteomic investigations, scientists could cross-correlate their findings and identify key proteins, the changes in the expression of which are most likely to be related to the schizophrenia neuropathogenesis. The later included proteins involved in metabolism, cytoskeleton, oxidative stress, synaptic and neuroprotective function. There was some overlap in protein candidates, but the majority of the altered proteins were region-specific [25, 26]. Furthermore, within the same regions of interest, the identified proteins were different when compared separately within the left or right hemisphere [26], and also within adjacent grey or white matter regions [25]. This fact prompted further proteomic investigations, e.g. how the grey and white matter proteomes differ from each other in normal controls [27].

Specific protein markers of the biological pathways involved in schizophrenia is another area where autopsy material is indispensable. Disturbances in cognition in schizophrenia, particularly in relation to motivation and attention, have attracted specific attention to the ACC. Receptor binding studies implicated imbalance of neurotransmitter regulation in schizophrenia, e.g. impaired endogenous cannabinoid system, glutamatergic and cholinergic muscarinic receptors in the ACC [28–33]. Furthermore, significantly reduced expression of human neuronal protein 22 (hNP22) in the ACC ndicates aberrant neuronal cytoarchitecture and dysregulation of neural signal transduction in schizophrenia [34].

The posterior cingulate cortex (PCC) in the limbic system is sensitive to MNDA receptor antagonists and is important for working memory. In schizophrenia, the PCC displays reduced metabolism and slow activation. As shown using the TRC cohorts, there are reductions in regional volumes and gyral folding of the PCC in schizophrenia [35]. A number of studies investigated this brain region by quantitative autoradiography. These studies showed abnormal expression of glutamate, GABA, muscarinic, and cannabinoid receptors, suggesting an increased acetylcholine and down regulated GABA stimulation in the PCC in schizophrenia [36–38]. The significant overlap of the cohorts used by these separate studies allows them to be compared directly. Furthermore, the use of the comprehensive clinical information available on antipsychotic drug treatments and cannabis use allows clinical correlations to be taken into account.

Pioneering investigations into the distribution of the tachykinin receptors, which play a role in inflammation and autonomic reflexes, have discovered increased neurokinin-1 receptor immunoreactivity in the prefrontal cortex (PFC) [39–41]. Despite the TRC providing the PFC and anterior hippocampus (HP) from carefully matched cohorts, a novel theory of reduced AKT-GSK3β molecular cascade signaling in schizophrenia was unable to be confirmed [42]. The same study did also show that phosphorylation levels of proteins in the postmortem brain can be correlated with PMI and tissue pH [42]. Calcium signaling has a vital role in neuronal signal transduction that is thought to be altered in schizophrenia. Other studies have been examining the contributions of specific calcium binding proteins (calbindin, calretinin and parvalbumin) to the molecular neuropathogenesis in the PFC and PCC of schizophrenia cases obtained from TRC cohorts [43, 44]. The superior temporal gyrus (STG) is a major anatomical substrate for speech, language and communication, all of which are disturbed in schizophrenia. By studying the STG, Deng and Huang [45, 46] elucidated molecular mechanisms that may be responsible for auditory hallucinations in schizophrenia. They reported an abnormal interaction between GABA receptors and various muscarinic receptors subtypes [45, 46].

The TRC has provided tissues for studies aiming to investigate changes in genes in schizophrenia. Altered gene expression in the STG in schizophrenia includes those involved in neurotransmission and neurodevelopment, and to a lesser extent presynaptic function [47]. Decreased STG expression of the regulator of G-protein signalling 4 (RGS4) mRNA in schizophrenia supports RGS4 as a potential genetic and functional biological marker of schizophrenia [48]. Furthermore, analysis of global miRNA expression in STG cortical grey matter reveals significant up-regulation of miR-181b expression in schizophrenia. This miRNA is predicted to regulate many target genes including calcium sensor gene visinin-like 1 and the ionotropic AMPA glutamate receptor subunit (GRIA2), both of which are found to be down-regulated in the same tissue [49]. A possible link between schizophrenia and NMDA-glycine site receptor ligand metabolic enzymes, D-amino acid oxidase and kynurenine aminotransferase-1 was demonstrated in cerebellum and parietal cortex using TRC cohorts [50].

Schizophrenia-related changes in the amygdala genome include genes involved in synaptic function, myelination and cellular signaling [51]. However, there is no amygdale-specific tachykininin-binding NK_1_ receptor contribution to the schizophrenia pathogenesis [52]. Further studies into molecular biological basis of schizophrenia and allied disorders using the TRC cohorts are warranted.

### 7.2. Alcohol use disorders

Research studies performed over the past ten years using the TRC tissue identified alcohol-responsive genes in different brain regions [53, 54]. The TRC study cohorts have included both non-cirrhotic and cirrhotic cases. Most recently, studies have also considered the combined effects with nicotine use.

Of 4000 target genes screened in the superior frontal cortex (SFC), 163 genes had altered expression in brain tissue from alcoholics [55]. These included genes associated with white matter, e.g. down-regulated myelin-related genes [56]. There is also down-regulation in the expression of genes involved in protein trafficking, cytoskeletal organization, plasma membrane recycling, protein sorting, synaptogenesis and synaptic plasticity [57]. The genes involved in calcium, cAMP, and thyroid signaling pathways are also altered in the brain due to alcohol abuse. The frontal and the motor cortex display widespread alterations in genes at transcriptional level [54]. A considerable alcohol-induced reprogramming of gene expression in the SFC occurs in genes not only involved in myelination and neurogenesis, but also in genes involved in ubiquitination, apoptosis, cell adhesion, and in neurodegenerative diseases, which suggests a link between alcoholism and other neurodegenerative conditions [58]. The neural adaptations to alcohol on cellular level also include an inhibition of protein and mRNA expression levels of intrinsic apoptotic pathways in the PFC in alcoholics [59], which reflects on the decreased cell density in this region [60, 61].

The mesocorticolimbic dopaminergic system, arising in the ventral tegmental area (VTA) and projecting to the nucleus accumbens (NA), the amygdala, the septum and the PFC, is a recognized reward pathway for drugs of abuse such as alcohol [53]. Examining this pathway, only 13 out of a total of 230 genes with altered expression were common between the NA and the PFC suggesting that alcoholism may be associated with adaptive changes specific to regions [62]. Further investigations showed alcohol-responsive genes in the NA and the VTA that primarily were associated with changes in neurotransmission and signal transduction [63]. A number of early gene expression profile studies in alcohol abuse did not consider the variable of nicotine use and this may explain the discrepancy in results of those using different cohorts [55, 57, 62]. However, long-term alcohol abuse, heavy smoking and co-abuse can have a selective impact on gene expression as has been demonstrated in the PFC and VTA regions [19, 20]. Consideration of different environmental and pathological conditions must be incorporated into the gene expression studies [19, 64, 65]. Complications of alcohol abuse such as liver cirrhosis are associated with widespread effects on the transcriptome of whole groups of genes (as compared with non-cirrhotic alcoholics), in particular genes involved in cell adhesion, mitochondrial function, synaptic transmission, apoptosis, and cell proliferation [66].

Neuroadaptations to the effects of chronic alcohol consumption include dysregulation of transcription factors of the NF-κB (Nuclear Factor-kappaB) family, which have implications for neuronal plasticity and neurodegeneration [67]. Elevated levels of synaptophysin I suggest a role in the enduring neuroplasticity in the prefrontal cortical glutamate circuitry that is associated with ethanol dependence [68].

On the proteome level, alcohol causes global changes in the expression of multiple proteins. In the superior frontal cortex, there were 182 proteins or protein isoforms with altered expression [69]. The majority of these (139) had lower expression levels in the alcoholic brain. In the PFC, 60 proteins change their expression characteristics in white matter with alcohol abuse compared with 44 identified in grey matter. The functional groups of the altered proteins included carbohydrate and protein metabolism, signal transduction/cell communication, cytoskeleton and homeostasis [70, 71]. Amongst changed proteins thiamine dependent enzymes transketolase and pyruvate dehydrogenase (E1ß subunit) are disturbed, even in “neurologically uncomplicated” alcoholics [72]. Cortical layers of the frontal cortex in alcoholics also showed increased levels of hNP22 protein, a cytoskeleton-interacting protein, which can be involved in morphological or plastic changes that are observed after chronic alcohol exposure and withdrawal [73]. An induction of two proteins, midkine and excitatory amino acid transporter 1, in the PFC of chronic alcoholics may reflect an adaptive reaction to accelerated neurodegeneration [19]. Proteomic investigations into the genu and splenium of the CC suggest that the mechanisms of alcohol-induced white matter damage may be different compared to the prefrontal region [74, 75]. Proteins involved in oxidative stress, lipid peroxidation and apoptosis were specific to cirrhotic alcoholics [75]. Another region that is highly alcohol sensitive is the hippocampus [76] with 17 different proteins showing alcohol-related altered expression.

### 7.3. Movement disorders and dementias

While movement disorders are not targeted by the TRC, brain and spinal cord donors who have Motor Neuron Disease (MND) are an exception. Using brain tissues provided by the TRC, researchers have excluded several possible causes of sporadic MND, including some viral infective agents [77], and impairment of methylation genes responsible for detoxifying the body from heavy metal exposure [78]. Blood samples of those with MND collected separately by the MND-DNA bank, in association with DNA obtained from brain tissues from the TRC, have expanded the types of studies able to be undertaken [79–81]. DNA studies from blood complement similar studies using brain tissues. More recent research in MND using TRC cases have been performed using microarray genotyping and other forms of gene analysis [82].

Parkinson’s disease is identified neuropathologically by the loss of pigmented neurons in the brainstem. These unique cells containing neuromelanin pigment are responsible for the black appearance of the substantia nigra in the brainstem. Studies using cases from the TRC and other sources have examined these dopaminergic pigmented neurons to ascertain the developmental stages of the neuromelanin pigment with normal aging [83, 84]. Such studies aid our basic understanding of human brain structure with age and in Parkinson’s disease.

A number of studies have utilized TRC control tissues in studies of Alzheimer’s disease (AD) and other dementias. An early study by Cullen et al [85] showed changes in astrocytes surrounding the microvasculature occurring with plaque formation. This gives an insight into how plaques are formed in AD. Other early studies examined cell losses in differing brain regions in AD, as for example, in the basal forebrain [86]. Apolipoprotein E (ApoE) s a well-known risk factor gene for AD, and relationships with ApoE status have been performed comparing cases with AD with control cases using TRC tissues [87]. The ApoE ɛ4 allele is associated with the deposition of β-amyloid known to be a major component of AD, especially for late-onset AD. More recent studies are examining other genes known to be associated with AD, including early onset forms [88, 89]. The inflammatory response in AD is another area of study of importance to dementia researchers. The TRC tissue collection protocols, whereby cases and controls are collected with short PMI and good agonal state, are extremely helpful for these types of studies. A recent study identified a number of upregulated cytokines and chemokines that mediate the inflammatory response in AD [89].

Frontotemporal dementia and Lewy body dementia are also important areas of study that have significant clinical and pathological overlap with other dementing diseases. The accurate neuropathological characterization of cases by the brain bank is an extremely important role. For example, cases with frontotemporal dementia with motor neuron disease inclusions need to be differentiated from cases with motor neuron disease without dementia. Similarly, cases with Lewy body dementia need to be differentiated from both AD (which has dementia) and from Parkinson’s disease (which doesn’t). Such clinical and pathological differentiation allows researchers to study these diseases on the molecular level. For example, tau deposition as part of the pathological presentation in frontotemporal dementia is not necessarily accompanied by altered tau gene expression [90].

## 8. Conclusions

The severe, debilitating nature and high worldwide prevalence of brain diseases imposes great personal, social, clinical and economic consequences, resulting in these illnesses being amongst the leading causes for disability globally [2]. Investigating the neurobiological basis of these diseases in the ultimate search for improved diagnostics and treatments requires access to well characterized and well preserved postmortem human brain tissue. The New South Wales Tissue Resource Centre in Sydney has a specific focus on psychiatric conditions and controls, and operates using best practice and quality control protocols. Since its establishment the TRC has become a key resource facility with an international reputation. Tissue provided by the TRC has enabled many independent studies to utilize novel molecular age technology. The protocols and operational procedures employed by the TRC accommodate the varied demands dictated by modern neuroscience. The TRC, in association with the pre-mortem brain donor programs and the Australian Brain Bank Network, holds great promise for the future.
